# Dual targeting non-overlapping epitopes in HER2 domain IV substantially enhanced HER2/HER2 homodimers and HER2/EGFR heterodimers internalization leading to potent antitumor activity in HER2-positive human gastric cancer

**DOI:** 10.1186/s12967-024-05453-8

**Published:** 2024-07-09

**Authors:** Ruicheng Wei, Wenli Zhang, Futang Yang, Qianhao Li, Qingyu Wang, Ningshu Liu, Jun Zhu, Yongqiang Shan

**Affiliations:** 1 Shanghai Henlius Biotech, Inc, Shanghai, 200233 China; 2 Global R&D Center, Shanghai Fosun Pharmaceutical (Group) Co., Ltd, Shanghai, 200233 China

**Keywords:** HLX22, HER2, Trastuzumab, Gastric cancer

## Abstract

**Background:**

Trastuzumab and pertuzumab combination has been approved for the treatment of patients with HER2-positive metastatic breast cancer. However, trastuzumab and pertuzumab combination did not show improvement in overall survival in patients with HER2-positive metastatic gastric cancer.

**Methods:**

We developed a new HER2-targeted monoclonal antibody, HLX22, targeting HER2 subdomain IV as trastuzumab but with non-overlapping epitopes. We examined the antitumor effects of this novel HER2-antibody in gastric cell lines and cell line-derived xenograft (CDX) and patient-derived xenograft (PDX) models.

**Results:**

HLX22 in combination with HLX02 (trastuzumab biosimilar) induced enhancement of HER2/HER2 homodimers and HER2/EGFR heterodimers internalization, which ultimately led to the reduction in signal transductions involving STAT3, P70 S6, and AKT; gene expressions of FGF-FGFR-PI3K-MTOR, EGF-EGFR-RAS, TGF-β-SMAD, PLCG and cell cycle progression related pathways that favor tumor development, proliferation, progression, migration and survival in gastric cancer cell line NCI-N87 were also reduced. These differing but complementary actions contributed to the synergistic antitumor efficacy of the HLX22 and HLX02 combination in gastric cancer cell lines, CDX and PDX. In addition, HLX22 in combination with HLX02 demonstrated stronger antitumor efficacy than HLX02 and HLX11 (a potential pertuzumab biosimilar) combination treatment both in vitro and in vivo.

**Conclusions:**

These results suggested that the application of non-competing antibodies HLX22 and HLX02 targeting HER2 subdomain IV together may be of substantial benefit to gastric cancer patients who currently respond suboptimal to trastuzumab therapy.

**Supplementary Information:**

The online version contains supplementary material available at 10.1186/s12967-024-05453-8.

## Background

Epidermal growth factor receptor (EGFR) family signaling contributes to neoplastic cell growth, malignant transformation, and resistance to chemotherapy [1]. Human epidermal growth factor receptor 2 (HER2) belongs to human EGFR family, including EGFR (HER1), HER3, and HER4. Trastuzumab, a monoclonal antibody targeting HER2, was introduced in clinical practice and revolutionized the treatment of HER2-positive breast cancer and gastric cancer. Despite this achievement, most patients with HER2-positive breast cancer and gastric cancer still showed disease progression, highlighting the need for new therapies. The continuous interest in novel targeted agents led to the development of pertuzumab. Trastuzumab binds to subdomain IV of HER2 extracellular domain, whereas pertuzumab binds to subdomain II of HER2 extracellular domain [2]. Trastuzumab inhibits the homodimerization of HER2 and the subsequent HER2/HER2 homodimer downstream signaling pathways, whereas pertuzumab preferentially blocks the heterodimerization of HER2 with EGFR, HER3, or HER4, and the subsequent HER2 heterodimers downstream signaling pathways [3, 4]. In addition, both pertuzumab and trastuzumab can also mediate antibody dependent cell-mediated cytotoxicity (ADCC) [1, 2].

U.S. Food and Drug Administration approved pertuzumab in combination with trastuzumab and docetaxel for the treatment of patients with HER2-positive metastatic breast cancer in 2013. And the combination regimen is now a standard of care in this indication. However, a phase III trial that assessed the efficacy of pertuzumab versus placebo in combination with trastuzumab and chemotherapy (capecitabine, 5-fluorouracil and cisplatin) in first-line HER2-positive metastatic gastric or gastro-esophageal junction cancer showed negative result[5]. These results suggest that there are intrinsic differences in the tumor biology of HER2-positive advanced gastric cancer and HER2-positive breast cancer. Several mechanisms have been proposed to explain the synergism of trastuzumab and pertuzumab in the treatment of HER2-positive cancers [6–9]. Several lines of evidence have suggested that in addition to inhibiting the HER2 homodimerization or HER2 heterodimerization with other EGFR family members and their corresponding downstream signaling pathways, the combination of trastuzumab and pertuzumab may also promote HER2 internalization [10–13]. Our preliminary study indicated that HLX02 or HLX11 alone induced only weak HER2 internalization; however, the combination of HLX02 and HLX11 resulted in enhanced HER2 internalization in human gastric cancer cell line NCI-N87 and human breast cancer cell line BT-474. These studies demonstrated that targeting non-overlapping epitopes in HER2 various domains to induce enhanced HER2 internalization and degradation seemed to be a promising strategy for HER2-targeted therapies.

To further enhance HER2 internalization, we developed a novel monoclonal antibody HLX22 targeting the subdomain IV of HER2 as trastuzumab, but acts on a distinct epitope. The combination of HLX22 and HLX02 enhanced HER2 internalization both in human gastric cancer cell line and in human breast cancer cell lines. Both in vitro and in vivo studies demonstrated that HLX22 in combination with HLX02 exhibited synergistic tumor growth inhibitory effect in gastric cancer cell lines, cell line-derived xenograft (CDX) and patient-derived xenograft (PDX) models, suggesting that gastric cancer patients may benefit from the HLX22 and HLX02 combination treatment. In addition, the phase I trial showed that HLX22 was well tolerated with favorable pharmacokinetic properties in patients with advanced HER2 overexpressing solid tumors, and no dose-limiting toxicity occurred during the study [14].

## Methods

### Antibodies

HLX22 is a humanized immunoglobulin G1 (IgG1) anti-HER2 monoclonal antibody. HLX02 is a trastuzumab biosimilar. HLX11 is a potential pertuzumab biosimilar targeting HER2.

### Structure simulation

The F(ab)’ structure of HLX22 was simulated by antibody modeling module in Molecular Operating Environment (MOE), and then docking with the crystal structure of trastuzumab and HER2 ECD complex (PDB code: 1N8Z) by protein–protein docking module.

### Cell lines

SKBR3, BT-474 and NCI-N87 were purchased from the National Cancer Institute. JIMT-1, SNU216, MKN45 and SNU16 were purchased from CoBioer (China). All cells were cultured in the recommended medium and supplemented with 10% fetal bovine serum (10091148, Gibco) at 37 ℃ with 5% CO_2_. All cell lines were free of mycoplasma. The identity of each cell line was verified via short tandem repeats (STR) analysis.

### Cell proliferation assay

Cell proliferation was quantified using the CellTiter-Glo Luminescent Cell Viability Kit (G7571, Promega). 0.5 × 10^4^–1.5 × 10^4^ cells were plated in 96-well plates (CLS3799, Corning), following overnight incubation at 37 ℃. The samples were treated with IgG1, HLX02, HLX22, HLX11, HLX02/HLX22 combination or HLX02/HLX11 combination. Meanwhile, luminescence values in the T = 0 h plates were measured. After incubated for 72 h, luminescence values in the T = 72 h plates were measured using Spark multimode reader (Tecan, Switzerland). The percentage cell viability was calculated: Viability = (T72–T0)/(T72(c)–T0) × 100%, T: treatment, c: Control. Combination effects were assessed by combination index (CI) and the detailed method was described in Liu et al. [15].

### ADCC and complement-dependent cytotoxicity assay

For the ADCC assay, the Jurkat-CD16a reporter assay was performed. Briefly, target cells (NCI-N87) were collected and counted, seeded at 1 × 10^5^/mL, 100 μl/well, into 96-well plates (Corning). Test antibodies were prepared from 3 μg/mL, 1:4 diluted 9 times. 50 μL diluted antibody solution was added to test wells, or 50 μL medium to the background control wells, or 50 μL IgG1 at 3 μg/mL to the isotype control wells. Effector cells (CD16a Jurkat) were collected and adjusted to 1 × 10^6^ cells/mL and 50 μL was added to test wells (the ratio of effector to the target cell was 5:1). The plate was incubated for 5 h in a humidified 5% CO2 atmosphere at 37 °C. After then, Bio-GloTM Luciferase Assay reagent (G7940, Promega) was added to each well, and relative luciferase units (RLU) was measured with Spark multimode reader (Tecan, Switzerland).

To evaluate complement-dependent cytotoxicity activity, CellTiter-Glo Luminescent Cell Viability Kit (G7573, Promega) was used. Briefly, target cells (NCI-N87) were washed in DPBS and resuspended in CDC buffer at a density of 12.5 × 10^4^ cells/mL, and then seeded at 5.0 × 10^3^ cells/well into 96-well plates (Corning). Normal human plasma was added to each well at a final dilution of 1:5, and then HLX02, HLX22, HLX11 and HLA-ABC (positive control) were added at a serial dilution of concentration (from 50 μg/mL to 1.6 × 10^−2^ μg/mL). The plates were centrifuged at 400 × *g* for 1 min and then incubated for 3 h in a humidified 5% CO2 atmosphere at 37 °C. The Luminescence values were detected as described above for cell proliferation assay. CDC for each sample is represented as Percent Specific lysis and was calculated as: % specific lysis = [(experimental Luminescence value /Mean of control Luminescence value)-1] × 100.

### Caspase activation assay

Activation of combined caspase-3 and caspase-7 was assessed using the Caspase-Glo^®^ 3/7 Assay (G8093, Promega). Approximately 1.0 × 10^4^–1.5 × 10^4^ cells were seeded in 96-well assay plates (CLS3799, Corning) and left to adhere overnight. Medium was removed and replaced with 100 µL medium containing drugs. Samples were incubated at room temperature for 30 min before adding 100 µL Caspase-Glo 3/7 reagent. Plates were protected from light and incubated for additional 30 min with gentle agitation and luminescence was measured using Spark multimode reader (Tecan, Switzerland).

### Human phospho-kinase proteome profiler analysis

The Human Phospho-Kinase Array Kit (ARY003C, R&D) was used to analyze the effects of different treatments on kinase phosphorylation. Each specific antibody was spotted in duplicate on the membranes, which also included appropriate controls. 3 × 10^6^ cells were seeded and treated with HLX02, HLX22, HLX11, HLX02/HLX11 combination or HLX02/HLX22 combination at 10 μg/mL for 4 h. Whole-cell lysates were extracted with cell lysis buffer supplemented with protease and phosphatase inhibitors according to the manufacturer’s instructions and a total protein amount of 600 μg were used in the Phospho-Kinase Array. Finally, unbound HRP antibodies were washed out and the signal detection was performed with Chemiluminescence detection system (CLiNX, Shanghai, China).

### RNA library preparation and sequencing

RNA-seq analysis was performed in NCI-N87, BT474 and SKBR3 cell lines following exposure to IgG1, HLX02, HLX22, HLX11, HLX02/HLX11 combination or HLX02/HLX22 combination at 10 μg/mL for 72 h. Cells were then lysed, and RNA extracted in TRIzol solution (15596026, Invitrogen). Long RNA libraries were prepared for sequencing using MGI library preparation kit and sequenced as PE150 on the BGISEQ Platform (BGI, China). The reads were mapped to the reference human genome sequence (hg38). The cancer-related genes sets were downloaded from KEGG (release 105.0) and employed to perform Gene Set Variation Analysis. ANOVA test was used to determine if there was significant variation among multiple groups. Pairwise comparison was conducted using *t* test (two groups).

### Flow cytometry

The EGFR, HER2 and HER3 protein expression levels were determined using BV421 Anti-Human EGF Receptor (749755, BD), AF647 anti-human CD340 (erbB2/HER-2) (324412, BioLegend), and BB700 Mouse Anti-Human ErbB3 (751796, BD). All stained cells were analyzed on CytoFLEX LX flow cytometer (Beckman Coulter, USA). Data were analyzed using FlowJo v10.8.1 software (Treestar Inc, USA) and GraphPad Prism 8.0 (GraphPad Software, USA).

### HER2 internalization assay

The pHrodo iFL Green STP ester amine-reactive dye (P35369, Invitrogen) was used in HER2 internalization assay according to the manufacturer’s protocol. NCI-N87, SNU216, BT474 or SKBR3 cells (1.5 × 10^5^ cells/well) were seeded in 96-well plates (CLS3799, Corning), and then incubated with HLX02, HLX22, HLX11, HLX02/HLX11 combination or HLX02/HLX22 combination (10 μg/mL) for 1 h on ice. After removal of unbound antibodies by briefly washing with washing buffer, a group of cells were then chilled on ice to stop the internalization, and the other groups were transferred to 37 ℃ for different periods of time. After incubation with antibodies, cells were fixed by fixation buffer (420801, BioLegend) for 30 min. Finally, all stained cells were washed with staining buffer and analyzed by CytoFLEX LX flow cytometer (Beckman Coulter, USA).

### siRNA knockdown of EGFR

A pool of two EGFR siRNAs (sense 1, 5′-UGUGGCUUCUCUUAACUCCU-3′, antisense 1, 5′- ACACCGAAGAGAAUUGAGGA -3′; sense 2, 5′-AGGAAUUAAGAGAAGCAACAUdTdT-3′, antisense 2, 5′-AUGUUGCUUCUCUUAAUUCCUdTdT-3′, ordered from AZENTA) were transfected into NCI-N87 by the Lipofectamine method (Lipofectamine RNAiMAX Transfection Reagent; Invitrogen, Carlsbad, CA). After transfection for 48 h, cells were put into 96-well plate in 4000/well and treated with HLX02 + HLX22 or control IgG for 72 h. Cell proliferation was quantified using the CellTiter-Glo Luminescent Cell Viability Kit (G7571, Promega).

### In vitro inhibition of patient-derived xenograft cells

Patient-derived xenograft cells (LD1-0017-200764 and LD1-0017-201149, provided by LIDE biotech) were isolated from PDX bearing mice, digested, washed, resuspended, and then seeded at 15000/well in 96-well plate, treated with titrated HLX02 + HLX22. After 6 days culture in 37 ℃ with 5% CO_2_, cell viability was quantified using the CellTiter-Glo Luminescent Cell Viability Kit (G7571, Promega).

### In vivo antitumor efficacy assay

In CDX model, BALB/c nude female mice received subcutaneous implantation with 5 × 10^6^ NCI-N87 cells per mouse. The mice were randomly assigned to groups when the mean tumor volume reached approximately 120–150 mm^3^ and then given the indicated treatment as described below once daily for a period of time. HLX22 or vehicle was administered twice a week by intraperitoneal injection (3, 10, 30 mg/kg, n = 8 mice per group) for 28 days. In another CDX study, HLX02, HLX22, HLX11, HLX02/HLX22 combination, HLX02/HLX11 combination and HLX11/HLX22 combination were administered twice a week by intraperitoneal injection (10 mg/kg, n = 8 mice per group) for 28 days. In PDX model, tumor xenografts were generated from gastric cancer tissue of patient and transplanted subcutaneously into the right back of each NCG mouse. HLX02, HLX02/HLX22 combination, and HLX02/HLX11 combination were administered twice a week by intraperitoneal injection in 0406022 PDX model (8 mg/kg, n = 5 mice per group) for 52–60 days. Tumor volumes and body weights were measured twice weekly ($${\text{tumor volume }} = {\raise0.7ex\hbox{$1$} \!\mathord{\left/ {\vphantom {1 2}}\right.\kern-0pt} \!\lower0.7ex\hbox{$2$}}\left( {{\text{length}}\, \times \,{\text{width}}^{{2}} } \right)$$ ).

### Statistical analysis

Statistical analyses were performed using GraphPad Prism 8.0 (GraphPad Software, USA). The data are presented as the mean $\pm$ SD for in vitro data or the mean $\pm$ SEM for in vivo data. Data were analyzed using unpaired two-sided *t* tests for in vitro and in vivo assays; GSVA (1.46.0) in R (4.2.2) was used for Gene Set Variation Analysis. ANOVA test was used to determine statistical significance from three or more treatment groups. Pairwise comparison was conducted using *t* test (two groups). Statistical significance was determined based on the* p* value: not significant (ns), *p* $>$ 0.05; *p $<$ 0.05; **p $<$ 0.01; ***p $<$ 0.001.

## Results

### HLX22 binds to HER2 subdomain IV without competing with HLX02 and shows comparable ADCC activity to HLX02

**Fig. 1:**
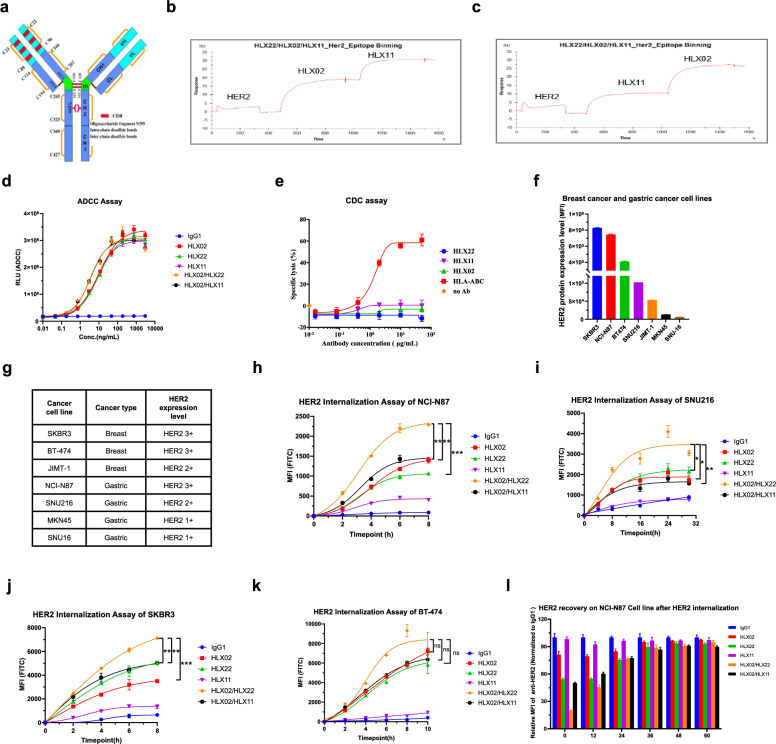
HLX22 in combination with HLX02 enhances HER2 internalization. **a** Structure of HLX22 monoclonal antibody. **b** and **c** HER2 epitope binding competing analysis of HLX22 with HLX02 and HLX11. **d** and **e** Analysis of HLX22 mediated antibody-dependent cell-mediated cytotoxicity and complement-dependent cytotoxicity activities. **f** and **g** HER2 protein expression levels in breast and gastric cell lines were evaluated and the relative expression level of HER2 are shown in the table. **h**–**k** Internalization time courses of HER2 antibody in NCI-N87, SNU216, BT-474 and SKBR3 cells stained with PHrodo Green dye. **l** Recovery of HER2 protein on NCI-N87 cells after removing HLX02, HLX22, HLX11, HLX02/HLX22 combination or HLX02/HLX11 combination treatment. Human IgG1 was used as negative control in **d**, **h**, **i**, **j**, **k** and **l**; HLA-ABC was used as positive control in **e**. The data are presented as the mean $\pm$ SD. ns: no significance, ***p < 0.001, **p < 0.01, *p < 0.05, using Student’s *t* test in **h**–**k**

HLX02 is a trastuzumab biosimilar, and HLX11 is a potential pertuzumab biosimilar. HLX22 is a humanized IgG1 anti-HER2 monoclonal antibody, which consists of two light chains and two heavy chains (Fig. 1a). Each light chain contains 214 amino acids and each heavy chain contains 443 amino acids, the CDR sequence are underlined (Additional file 1: Fig. S1). HLX22 has been confirmed to have a different binding site from HLX02 and binds to HER2 subdomain IV without competing with HLX02 from epitope binning (Fig. 1b, c). We performed antibody modeling and protein–protein docking analysis with MOE, based on the in-vitro data obtained from experiments, one of the structures that best fits the experimental results has been selected (Additional file 1: Fig. S2a). The protein interaction analysis showed that G110 in heavy chain plays an important role in antigen binding, D56 and W32 in light chain CDR2 are also important in antigen binding (Additional file 1: Fig. S2b). The key residues for HLX22 antibody binding on HER2 ECD are R523, H490 and Q526, which is different from trastuzumab including D560, E558 and K593 (figure S3a). Human gastric cancer cell line NCI-N87 was used to examine the ADCC activity of HLX22. These results showed that HLX22 and the comparator drug HLX02 and HLX11 all had ADCC activity and that the ADCC activity was comparable among these antibodies (Fig. 1d). However, neither HLX22 and HLX02 combination nor HLX02 and HLX11 combination shown enhanced ADCC activity compared to their single agent counterparts. HLX22, HLX02 or HLX11 did not show complement-dependent cytotoxicity activity on HER2-positive NCI-N87 cells (Fig. 1e).

### HLX22 in combination with HLX02 effectively enhances HER2 internalization

To study the internalization kinetics of HLX22 and HLX02 combination, human gastric cancer cell line NCI-N87 and SNU216, as well as human breast cancer cell line BT-474 and SKBR3, were bound with HLX22, HLX02, HLX11, HLX22 and HLX02 combination, or HLX02 and HLX11 combination. After 8 h, HER2 binding with HLX11 showed limited HER2 internalization in the HER2 3 + cell line NCI-N87, BT-474 and SKBR3 (Fig. 1f–k). The cells treated with HLX22 or HLX02 alone showed increased HER2 internalization to similar extent. Remarkably, HLX22 and HLX02 simultaneously bound to HER2 subdomain IV and induced much stronger HER2 internalization by 40–80% in these cells. In contrast, HLX02 and HLX11 simultaneously bound to HER2 subdomain IV and subdomain II, respectively, did not enhance HER2 internalization. In NCI-N87 cell line, when HLX22 and HLX02 were removed from supernatant, HER2 expression recovered completely 40 h after internalization (Fig. 1l). In HER2 2 + gastric cancer cell line SNU216, it took 24 h to reach HER2 internalization plateau; HLX22 and HLX02 combination, but not HLX02 and HLX11 combination, enhanced HER2 internalization.

### HLX22 in combination with HLX02 promotes HER2/HER2 homodimer internalization and HER2/EGFR heterodimer internalization

**Fig. 2:**
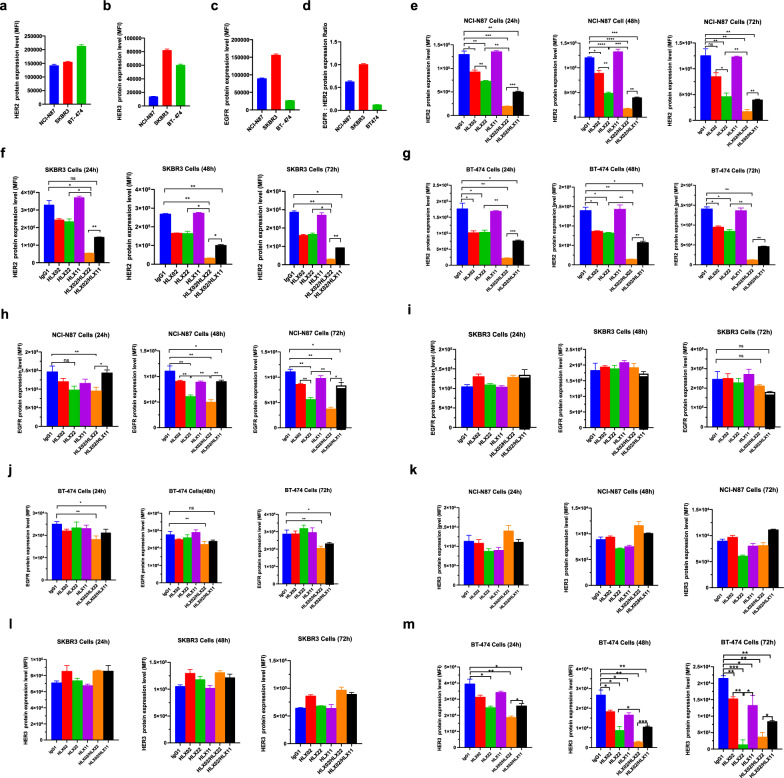
Enhanced HER2 internalization in gastric cancer cell line leads to downregulation of EGFR cell surface expression. **A**–**d** HER2, HER3 and EGFR expression status of gastric cancer cell line NCI-N87 or breast cancer cell line SKRB3 and BT-474. **e**–**g** Change of HER2 protein in NCI-N87 (**e**), SKBR3 (**f)** or BT-474 (**g**) cells for 24 h, 48 h or 72 h treatment. **H**–**j** Change of EGFR protein in NCI-N87 (**h**), SKBR3 (**i**) or BT-474 (**j**) cells for 24 h, 48 h or 72 h treatment. **K**–**m** Change of HER3 protein in NCI-N87 (**k**), SKBR3 (**l**) or BT-474 (**m**) cells for 24 h, 48 h or 72 h treatment. The data shown are representative of three biological replicates and are presented as the mean $\pm$ SD. ns: no significance, ***p < 0.001, **p < 0.01, *p < 0.05, using Student’s *t* test in **e**–**m**

Upon ligand binding, the EGFR family members can homodimerize or heterodimerize with each other to form several receptor complexes. The dimerization of EGFR family members results in protein phosphorylation on their intracellular domain that activates intracellular signaling cascades, which ultimately promote the expression of target genes that regulate various cellular processes influencing growth, proliferation, migration and survival [3, 4]. Previous studies have reported that breast cancer expresses a little higher level of HER2 and HER3 than gastric cancer [16, 17]. Analysis of The Cancer Genome Atlas datasets showed that the HER2 median mRNA expression level in breast cancer was 112.54 TPM, and the HER2 median mRNA expression level of gastric cancer was 65.35 TPM (Additional file 1: Fig. S3a). In addition, breast cancer also expressed a little higher level of HER3 than gastric cancer; the median mRNA expression level was 115.57 TPM and 87.21 TPM, respectively (Additional file 1: Fig. S3b). However, gastric cancer expressed much higher level of EGFR than breast cancer with a median mRNA expression level of 13.11 TPM, whereas breast cancer expressed very low level of EGFR, with a median mRNA expression level of 3.03 TPM (Additional file 1: Fig. S3c). 2 case reports also demonstrated that gastric cancer expressed higher level of EGFR than breast cancer [18], and it has been reported that high expression of EGFR predicts poor survival in patients with resected T3 stage gastric adenocarcinoma [19]. Gastric cancer cell line NCI-N87 and breast cancer cell line SKRB3 and BT-474 are representative of gastric cancer and breast cancer regarding HER2, HER3 and EGFR expression status (Fig. 2a–c).

Gastric cancer cell line NCI-N87 expressed high level of HER2 and EGFR (Fig. 2a, c), and HER2 and EGFR cell surface level were very close (Fig. 2d), indicating that these EGFR and HER2 had great chance to exist on cell surface in the form of HER2/EGFR heterodimer upon ligand bindings. Indeed, HLX22 and HLX02 that directly bound to HER2 in NCI-N87 cells not only decreased HER2 expression but also decreased EGFR expression on cell surface, suggesting that EGFR was internalized together with HER2 in the form of HER2/EGFR heterodimer (Fig. 2e, h). Due to the low HER3 expression, no decrease in HER3 cell surface expression was observed in NCI-N87 cells treated with HLX22 and HLX02 combination (Fig. 2b, k). Similarly, HER2 and HER3 cell surface expression levels were very high in BT-474 cells (Fig. 2a, b), and HER3 cell surface expression decreased in BT-474 cells treated with HLX22 and HLX02 combination, also suggesting that HER3 was internalized together with HER2 in the form of HER2/HER3 heterodimer (Fig. 2g, j, m). Among the above mentioned 3 cell lines, EGFR and HER3 cell surface expression levels were the highest in SKRB3 cells, but HER2 expression was not as high as BT-474 (Fig. 2a–c). Thus, there was not enough number of HER2 to promote the formation of HER2/EGFR heterodimers and HER2/HER3 heterodimers, and consequently, HLX22 and HLX02 could not efficiently decrease EGFR and HER3 cell surface expression in SKRB3 cells (Fig. 2f, i, l).

In contrast, pertuzumab disrupted the formation of HER2 heterodimer. Therefore, HLX02 and HLX11 combination disrupted HER2/EGFR heterodimer formation, and consequently, HLX02 and HLX11 combination only promoted HER2 internalization but could not promote EGFR internalization together in gastric cancer cell lines; EGFR still transduced signals that promoted tumor growth (Fig. 2h). This might be one of the reasons why trastuzumab and pertuzumab combination failed in gastric cancer clinical trial. To further investigate the involvement of EGFR in the efficacy of HLX22, we performed siRNA-EGFR in N87 cells, the results showed that the combination effects of HLX02 and HLX22 were significantly reduced (Additional file 1: Fig. S4a). Besides, in two in vitro patient-derived tumor models, we found that the combination effects of HLX02 and HLX22 are stronger in model with high EGFR expression compared with model with low EGFR expression (Additional file 1: Fig. S4b-c). Together, these data suggest that EGFR expression level could be a potential biomarker for patient stratification in the future.

### HLX22 in combination with HLX02 synergistically inhibits tumor cell growth in vitro

**Fig. 3:**
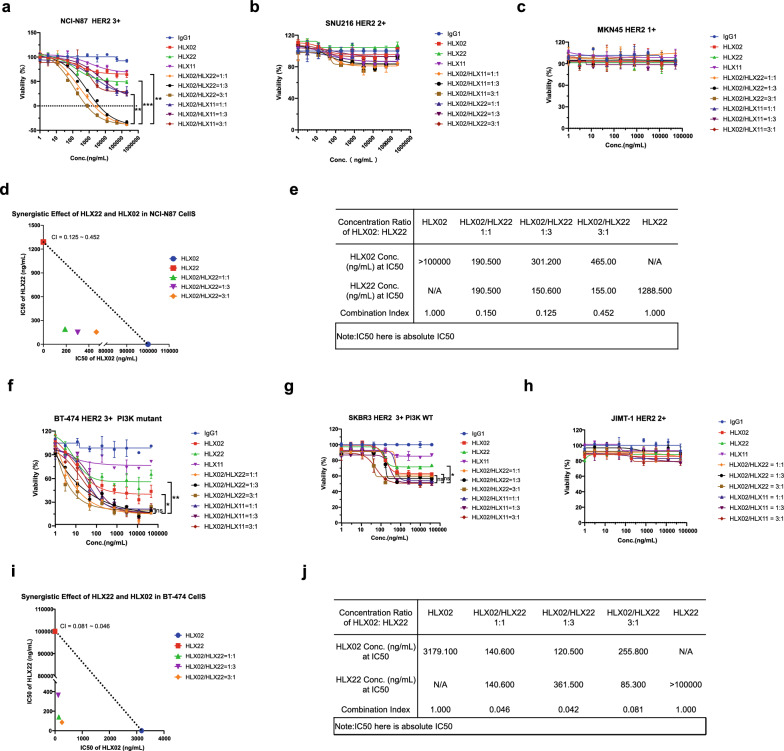
HLX22 in combination with HLX02 exhibits synergistic anti-tumor cell proliferation in HER2 3 + gastric cancer cell line in vitro. **a**–**c** Dose–response curves showing the cell viability assay of gastric cancer cell lines NCI-N87 (**a**), SNU216 (**b**) and MKN45 (**c**) treated with HLX02, HLX22, HLX11, HLX02/HLX22 combination or HLX02/HLX11 combination for 72 h. **d** and **e**, IC50 isobolograms of HLX02 and HLX22 combination were generated using a 72 h CellTiter-Glo proliferation assay. Combination effects were assessed using combination index (CI) and isobolograms for NCI-N87 cells. **f**–**h** Dose–response curves showing the cell viability assay of breast cancer cell lines BT-474 (**f**), SKBR3 (**g**) and JIMT-1 (**h**) treated with HLX02, HLX22, HLX11, HLX02/HLX22 combination or HLX02/HLX11 combination for 72 h. **i** and **j** IC50 isobolograms of HLX02 and HLX22 combination were generated using a 72 h CellTiter-Glo proliferation assay. Combination effects were assessed using CI and isobolograms for BT-474 cells. The data shown are representative results for two independent experiments and dose–response curves are presented as the mean $\pm$ SD. It is defined that CI < 0.3, 0.3 < CI < 0.6, and 0.6 < CI < 0.9 refer to very strong, strong, and moderate synergy, respectively. ns: no significance, ***p < 0.001, **p < 0.01, *p < 0.05, using Student’s *t* test in **a**, **f** and **g**

The synergistic action of trastuzumab and pertuzumab combination treatment was previously shown in vitro [20] using the calculation of the combination index as outlined by Chou and Talalay [15, 21, 22]. HLX22 or HLX02 alone inhibited the growth of NCI-N87 in vitro (3 + HER2 expression). In combination with HLX02, HLX22 could potentiate its anti-proliferation activity, inducing − 26.49% relative cell viability rate at 12.5 μg/mL (Fig. 3a). Whereas, HLX22 or HLX02 alone at 12.5 μg/mL resulted in 55.6% and 71.72% relative cell viability rate, respectively (Fig. 3a). Combination of HLX22 and HLX02 led to very strong synergistic inhibitory effects on tumor cell proliferation with combination indices ranging from 0.125 to 0.452, indicating very strong synergy (Fig. 3d, e). In addition, the combination of HLX22 and HLX02 displayed stronger tumor cell growth inhibitory effect than the combination of HLX02 and HLX11 (Fig. 3a). In human gastric cancer cell lines that HER2 expression levels were lower than 3 + , such as SUN216 (2 + HER2 expression) and MKN-45 (1 + HER2 expression), HLX22 in combination with HLX02 could not potentiate its anti-proliferation activity (Fig. 3b, c). This observation was further confirmed by a synergistic inhibitory effect of HLX22 and HLX02 on tumor cell proliferation in human breast cancer cell lines. The combination of HLX22 and HLX02 also led to strong synergistic inhibitory effects on BT-474 breast cancer cell proliferation with combination indices ranging from 0.081 to 0.046 (strong synergy, Fig. 3f, i, j), as well as on SKRB3 breast cancer cell proliferation (3 + HER2 expression, Fig. 3g). HLX22 in combination with HLX02 could not enhance anti-proliferation activity in JIMT-1 breast cancer cells (2 + HER2 expression) (Fig. 3h). Collectively, these data demonstrated that the combination of HLX22 and HLX02 induced synergistic inhibitory effects on tumor cell proliferation in tumor cells with 3 + HER2 expression.

### HLX22 in combination with HLX02 strongly enhances antitumor activity in human gastric CDX and PDX tumor models with 3 + HER2 expression

**Fig. 4:**
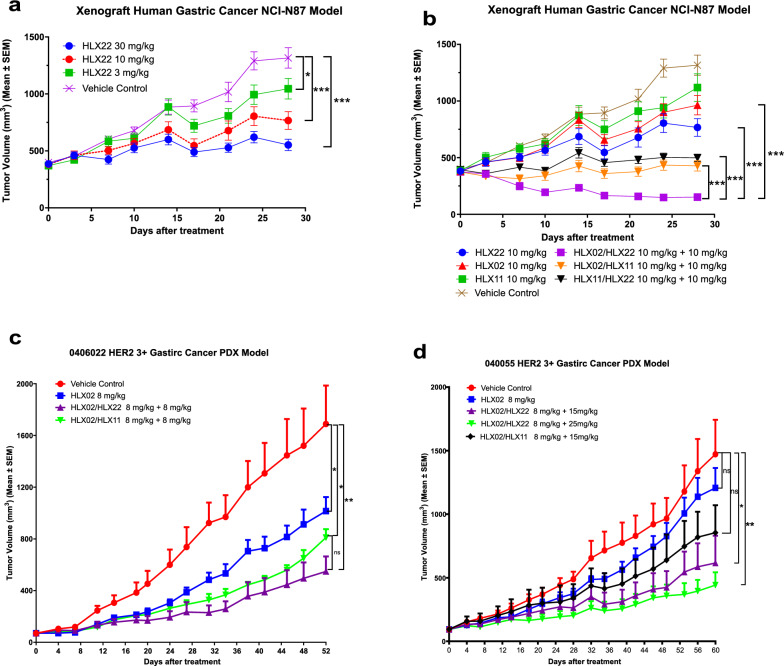
HLX22 in combination with HLX02 induces synergistic anti-tumor efficacy in HER2 3 + xenograft human gastric cancer NCI-N87 model and human gastric cancer PDX models.** a** and **b** Tumor volume curves for xenograft human gastric cancer NCI-N87 models treated with the indicated antibody at the indicated doses. **c** and **d** Tumor volume curves for human gastric cancer PDX models treated with the indicated antibody at the indicated doses. All data are presented as mean $\pm$ SEM. The* p* values (**a**–**d**) were calculated using Student’s *t* test. ns: no significance, ***p < 0.001, **p < 0.01, *p < 0.05

To further confirm this synergistic antitumor activity, we investigated the antitumor efficacy of HLX22 in combination with HLX02 in human gastric cancer cell NCI-N87 tumor xenografts. Tumor-bearing mice were injected with HLX22 at different doses (30 mg/kg, 10 mg/kg and 3 mg/kg), twice weekly, for 4 consecutive weeks. High-dose and medium-dose HLX22 demonstrated significant inhibition of NCI-N87 tumor growth compared to the vehicle control (*p* < 0.001) (Fig. 4a). HLX22 and HLX02 combination produced synergistic antitumor effect, with a better tumor growth-inhibiting effect than either HLX22 or HLX02 alone (Fig. 4b). In addition, HLX22 in combination with HLX02 produced a better tumor growth-inhibiting effect than the combination of HLX02 and HLX11. To further validate this observation, 2 human gastric cancer PDX models with 3 + HER2 expression were applied to evaluate the synergistic antitumor efficacy of HLX22 and HLX02 combination. HLX22 in combination with HLX02 exhibited significant stronger antitumor efficacy compared to their single agent counterparts (Fig. 4c, d). Furthermore, the antitumor efficacy of HLX22 and HLX02 combination therapy was greater than that of HLX02 and HLX11 combination therapy. Collectively, in line with the in vitro results, HLX22 in combination with HLX02 strongly enhanced antitumor activity either in human gastric CDX or in PDX tumor models with 3 + HER2 expression, suggesting that HLX22 and HLX02 combination regimen had the potential to change the paradigm for treatment of HER2-positive gastric cancer. The strongly enhanced antitumor activity of HLX22 and HLX02 combination may result from the enhancement of HER2/HER2 homodimers and HER2/EGFR heterodimers internalization. The enhancement of HER2/HER2 homodimers and HER2/EGFR heterodimers internalization may lead to reduction in signal transductions and gene expressions that favor tumor development, proliferation, progression, migration and survival.

### HLX22 in combination with HLX02 specifically enhances apoptotic cell death in NCI-N87 cells in vitro

**Fig. 5:**
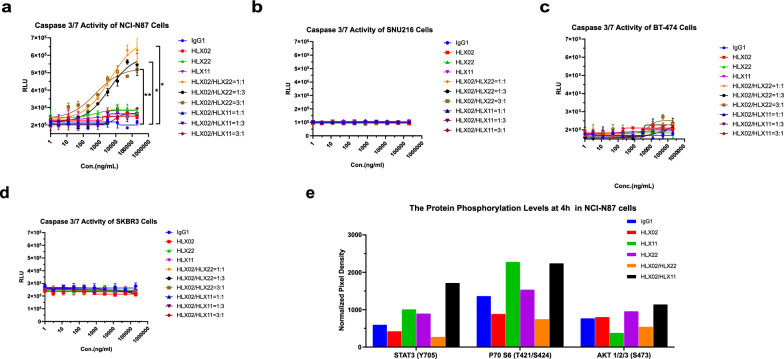
HLX22 in combination with HLX02 induces inhibition of cellular signal transduction and enhances apoptotic effect of human gastric cancer cell NCI-N87 with HER2 3 + expression. **a**–**d** Apoptosis was evaluated by measuring activation of the effector caspases 3/7 using Caspase-Glo^®^ 3/7 Assay systems in NCI-N87 (**a**), SNU216 (**b**), BT-474 (**c**), and SKBR3 (**d**) cells treated with the indicated antibody at the indicated doses. Analysis of proteome human Phospho-Kinase in NCI-N87 cells (**e**) with the indicated antibody for 4 h. The data are representative results for two independent experiments and dose–response curves are presented as the mean $\pm$ SD in **a**, **b**, **c** and **d**. Data are expressed as mean of duplicate spots in **e**. ns: no significance, ***p < 0.001, **p < 0.01, *p < 0.05, using Student’s *t* test in **a**

To identify the mechanisms of action for the synergistic antitumor activity of HLX22 and HLX02, we next performed mechanism analysis. HLX22 in combination with HLX02 induced − 26.49% relative cell viability rate at 12.5 μg/mL in NCI-N87 cells (Fig. 3a). This relative cell viability rate was lower than 0%, which suggested that HLX22 in combination with HLX02 not only inhibited tumor cells proliferation but also promoted cell death. Thus, we first evaluated apoptotic activity induced by HLX22 in combination with HLX02 in gastric cancer cell line NCI-N87 and SNU-261, as well as breast cancer cell line BT-474 and SKRB3. In gastric cancer cell line NCI-N87 with 3 + HER2 expression, limited caspase 3/7 activity was observed in cells treated with HLX22, HLX02 or HLX11 as single agent. Whereas, HLX22 in combination with HLX02 led to very strong caspase 3/7 activity (Fig. 5a). In contrast, the combination of HLX02 and HLX11 induced little caspase 3/7 activity (Fig. 5a). However, the combination of HLX22 and HLX02 did not induced caspase 3/7 activity on SUN216 cells with 2 + HER2 expression (Fig. 5b). Strikingly, neither HLX22 in combination with HLX02 nor HLX02 in combination with HLX11 induced caspase 3/7 activity in breast cancer cell line BT-474 and SKRB3 (Fig. 5c, d). PI3K mutations in breast cancer have been reported to associate with anti-apoptosis [23], but the failure to induce apoptosis may not be associated with PI3K mutation status, as BT-474 is PI3K mutant and SKRB3 is PI3K wild type. These results suggested that HLX22 in combination with HLX02 preferentially promoted apoptotic cell death in gastric cancer cells with 3 + HER2 expression compared to breast cancer cells with 3 + HER2 expression, implying that there were intrinsic differences in the tumor biology between HER2-positive gastric cancer and HER2-positive breast cancer in response to dual HER2 blockade.

### HLX22 in combination with HLX02 intervenes various cellular processes in protein signal transduction level to orchestrate antitumor efficacy

To understand how cells recognize and respond to changes in their environment upon HLX22 and HLX02 combination treatment, a Proteome Profiler Human Phospho-Kinase array, detecting the relative levels of phosphorylation of 43 kinase phosphorylation sites, was performed using gastric cancer cell line NCI-N87. A large volume of published studies have described the role of protein signal transduction on tumorigenesis and tumor progression previously. PI3K-AKT signal transduction inhibits tumor cell death through PI3K-AKT phosphorylation [24]. P70 S6K phosphorylation transduces anti-apoptosis signal via down regulating the expression of pro-apoptotic factor BAD [24]. Constitutive STAT3 activation was found in HER2-positive tumors and associated with tumorigenesis and drug resistance [24–28]. The phosphorylation of STAT3 (Y705), p70P70 S6 (T421/S424), and AKT1/2/3 (S473) was detected in IgG1-treated cells (Fig. 5e). HLX02 treatment resulted in a slight reduction in phosphorylated STAT3 (Y705) and P70 S6 (T421/S424) but did not alter the phosphorylation level of AKT1/2/3 (S473) compared to IgG1 treatment (Fig. 5e). Cells treated with HLX22 showed an increase in STAT3 (Y705), P70 S6 (T421/S424) and AKT1/2/3 (S473) phosphorylation levels compared to the IgG1-treated cells. The combination of HLX22 and HLX02 resulted in a dramatic decrease in STAT3 (Y705), P70 S6 (T421/S424), and AKT1/2/3 (S473) phosphorylation levels, suggesting that HLX22 in combination with HLX02 could effectively inhibit cancer cell proliferation, induce cancer cell apoptosis and prevent drug resistance. Strikingly, the combination of HLX02 and HLX11 dramatically increased STAT3 (Y705), P70 S6 (T421/S424), and AKT1/2/3 (S473) phosphorylation levels, which potentially promoted tumor cell proliferation, anti-apoptosis and drug resistance in NCI-N87 gastric cancer cells. These finding partially explained why trastuzumab plus pertuzumab and chemotherapy failed in the previously mentioned phase III clinical trial for the treatment of HER2-positive advanced gastric cancer.

### HLX22 in combination with HLX02 intervenes various cellular processes in gene transcription level to orchestrate antitumor efficacy

**Fig. 6:**
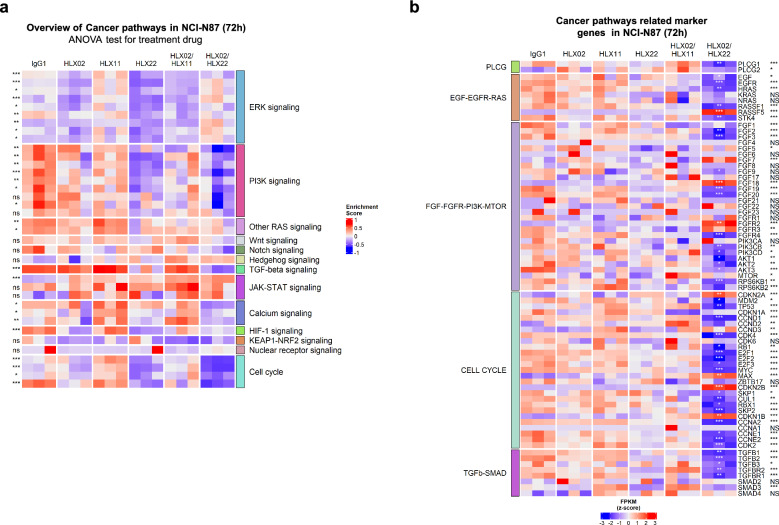
HLX22 in combination with HLX02 downregulates genes involved in FGF-FGFR-PI3K-MTOR, EGF-EGFR, TGF-beta-SMAD, and PLCG pathways in NCI-N87 cells. Heatmap showing unsupervised clustering of gene expression signatures and enrichment of cancer related pathways altered in NCI-N87 cells (**a**) treated with HLX02, HLX22, HLX11, HLX02/HLX22 combination, and HLX02/HLX11 combination (10 μg/ml) for 72 h. Individual treatments are shown in columns and annotated by cancer related pathways. ANOVA was used to determine statistical significance. ns: no significance, ***p < 0.001, **p < 0.01, *p < 0.05. Heatmap of gene expression of cancer related pathways altered in HLX02/HLX22 combination treatment compared to IgG1, HLX02, HLX11, HLX22, and HLX02/HLX11 treatments in NCI-N87 cells (**b**). Pairwise comparison between HLX22 and HLX02 combination treatment and HLX02 treatment was conducted using *t* test (***p < 0.001, **p < 0.01, *p < 0.05; labeled inside the column). ANOVA test was used to determine statistical significance from three or more treatment groups (ns: no significance, ***p < 0.001, **p < 0.01, *p < 0.05; labeled on the right side of the figure)

To investigate the modulatory effect of HLX22 in combination with HLX02 on gene expression profile in gastric cancer cell line with 3 + HER2 expression, we treated NCI-N87 cells with HLX22, HLX02, HLX11, HLX22 in combination with HLX02, or HLX02 in combination with HLX11 for 72 h and then examined the whole transcriptome by RNA-sequencing. Gene Set Variation Analysis showed that HLX22 in combination with HLX02 decreased the expression of PI3K signaling, RAS signaling, TGF-β signaling, Calcium signaling, HIF-1 signaling and cell cycle progression related pathways compared to the other treatment arms (Fig. 6a).

FGF-FGFR-PI3K-MTOR signaling pathways are commonly deregulated in cancers and promote cellular growth, proliferation, and survival [29]. Compared to the other treatment arms, HLX22 and HLX02 combination-treated cells showed lower expression levels of *FGF2*, *FGF3*, *FGF9*, *FGF19*, *FGF20*, *FGFR4*, *PI3KCB*, *PI3KCD*, *AKT1*, *AKT2*, *AKT3* and *RPS6KB1*, but showed higher expression levels of *FGF18* and *FGFR2* (Fig. 6b). Although FGF ligand *FGF18* and FGFR receptor *FGFR2* were upregulated, their downstream genes *PI3KCB*, *PI3KCD*, *AKT1*, *AKT2*, *AKT3* and *RPS6KB1* were downregulated, which suggested that HLX22 in combination with HLX02 inhibited cellular growth, proliferation, and survival through efficiently inhibiting the FGF-FGFR-PI3K-MTOR signaling pathway (Fig. 6b). The EGFR and RAS/RAF signaling pathway plays pivotal roles in tumor progression via proliferation, survival, invasion, and immune evasion [30]. The expression levels of EGF-EGFR-RAS signaling marker genes *EGF*, *EGFR*, *HRAS*, *RASSF1* and *STK4* in the HLX22 and HLX02 combination-treated cells decreased, suggesting that HLX22 in combination with HLX02 inhibited EGF-EGFR-RAS signaling to suppress tumor proliferation, survival, and invasion (Fig. 6b). TGF-β is a proinflammatory cytokine known to promote tumor development and mediate metastatic progression and chemoresistance during the later stages of tumor progression [31]. HLX22 and HLX02 combination-treated cells showed lower expression levels of TGF-β-SMAD signaling marker genes *TGFB1*, *TGFB2*, *TGFB3*, *TGFBR1* and *TGFBR2*, which suggested that HLX22 in combination with HLX02 inhibited TGF-β-SMAD signaling to inhibit the metastasis of tumor cells and overcome chemoresistance (Fig. 6b). PLCG is an essential mediator of cellular signaling. PLCG regulates multiple cellular processes via calcium (Ca^2+^) mobilization and activation of protein kinase C, other kinases and ion channels, and PLCG is frequently enriched and mutated in various cancers, and is involved in the processes of tumorigenesis, including proliferation, migration, and invasion [32].The expression levels of PLCG signaling marker genes *PLCG1* in the HLX22 and HLX02 combination-treated cells decreased, suggesting that HLX22 in combination with HLX02 inhibited PLCG signaling to restrain tumor proliferation, migration, and invasion (Fig. 6b).

HLX22 and HLX02 combination-treated cells downregulated the expression of *CCNA2*, *CCND1*, *CCNE1*, *CCNE2*, *CDK2*, *CDK4*, *SKP1*, *SKP2*, *CUL1*, *E2F1*, *E2F2* and *E2F3* genes that promoted G1/S transition and *CCNA1* gene that promoted G2/M transition compared to IgG1-treated cells (Fig. 6b). Meanwhile, *CDKN1B*, *CDKN2A* and *CDKN2B* genes that inhibited cell cycle progression were upregulated in HLX22 and HLX02 combination-treated cells (Fig. 6b). *MDM2* and *MYC* were also downregulated in HLX22 and HLX02 combination-treated cells. Taken together, these data indicated that HLX22 in combination with HLX02 induced tumor cell cycle arrest by downregulating G1/S transition and G2/M transition genes and upregulating cell cycle repressor genes. Whereas, cells treated with HLX02 and HLX11 combination did not display any downregulation of G1/S transition and G2/M transition genes or upregulation of cell cycle repressor genes.

Collectively, these results suggested that enhancement of HER2/HER2 homodimers and HER2/EGFR heterodimers internalization led to down-regulation of gene expressions involving pathways that favor tumor development, proliferation, progression, migration and survival in gastric cancer cell line NCI-N87.

## Discussion

Trastuzumab in combination with pertuzumab and docetaxel has been approved for the treatment of patients with HER2-positive metastatic breast cancer, but trastuzumab in combination with pertuzumab and chemotherapy in the first-line treatment of HER2-positive metastatic gastric or gastro-esophageal junction cancer showed negative results in a phase III trial [5, 33], suggesting that there are intrinsic differences in the tumor biology of HER2-positive advanced gastric cancer and HER2-positive breast cancer in response to HER2 dual blockade. Although both breast cancer and gastric cancer highly express HER2, gastric cancer expresses higher level of EGFR than breast cancer. 40.6% of human gastric cancer samples exhibited high expression of EGFR (IHC score, 3 +) and 59.4% of human gastric cancer samples exhibited low expression of EGFR (1 + and 2 +) [19]. Whereas, 11.8% of human breast cancer samples exhibited high expression of EGFR (3 +) and 25.4% of human breast cancer samples exhibited low expression of EGFR (1 + and 2 +), and 62.9% of human breast cancer samples did not express EGFR protein (IHC score 0) [18]. Moreover, it has been reported that high expression of EGFR predicts poor survival in patients with resected T3 stage gastric adenocarcinoma [19]. There are also intrinsic differences in the mechanisms of action between trastuzumab and pertuzumab. Trastuzumab interferes with HER2 signaling via several mechanisms: stimulation of HER2 endocytosis, inhibition of the downstream proliferation pathways and promoting apoptosis [1]. Whereas, pertuzumab binds to the dimerization domain (extracellular domain II) of HER2, resulting in inhibition of ligand-induced HER2 homodimerization or heterodimerization, which leads to blockade of HER2 downstream signaling pathways [3, 4]. These complexities in tumor biology and antibody mechanisms of action severely hamper the application of trastuzumab and pertuzumab combination therapy.

Our findings indicated that HLX02 and HLX11 combination disrupted HER2/EGFR heterodimer formation, and consequently, HLX02 and HLX11 combination only induced HER2/HER2 homodimers internalization, but could not promote HER2/EGFR heterodimers internalization on gastric cancer cells, and therefore EGFR kept transducing signals that promoted tumor growth (Fig. 2e, h). In contrast, the combination of HLX22 and HLX02 not only enhanced HER2/HER2 homodimers internalization, but also induced HER2/EGFR heterodimers internalization both in HER2 3 + gastric cancer cell lines and in HER2 3 + breast cancer cell lines, which inhibited tumor growth promoting signals to a greater extent (Fig. 2e, h). Consequently, this unique HER2-targeted strategy demonstrated stronger synergistic tumor growth inhibitory effect in gastric cancer cell lines (Fig. 3a–e), CDX models (Fig. 4a, b) and PDX models with 3 + HER2 expression compared to HLX02 and HLX11 combination treatment (Fig. 4c, d).

The strongly enhanced antitumor activity of HLX22 and HLX02 combination was likely attributable to their differing but complementary mechanisms of action in protein signal transduction levels and gene transcription levels in response to the enhancement of EGFR receptor family internalization. It has been reported that the EGFR receptor family dimerization and phosphorylation lead to the activation of intracellular signaling cascades, and ultimately, these signaling cascades lead to the expression of target genes that regulate various cellular processes that favor tumor growth, proliferation, migration and survival [34]. In this study, HLX22 in combination with HLX02 regulated various cellular processes regarding anti-proliferation, induction of apoptosis, inhibition of tumor migration and prevention of drug resistance through intervening the EGFR receptor family related protein signal transductions, as well as down-regulation of gene expressions involving FGF-FGFR-PI3K-MTOR, EGF-EGFR-RAS, TGF-β-SMAD, PLCG, and cell cycle progression related pathways, which ultimately orchestrated the synergistic antitumor efficacy. HLX22 and HLX02 combination did not exhibit enhanced ADCC activity compared to their single agent counterparts in vitro (Fig. 1d), suggesting that enhanced ADCC was unlikely to be the mechanism by which the strongly enhanced activity of HLX22 and HLX02 combination, observed in vivo, was mediated. However, ADCC may still provide a mechanism by which HLX22 and HLX02 combination may also provide an antitumor effect.

## Conclusions

In conclusion, HLX22 in combination with HLX02 showed a strongly enhanced antitumor effect and promoted tumor regression in HER2-positive human gastric cancer CDX models and PDX models. Both in vitro and in vivo studies demonstrated that the antitumor activity of HLX22 and HLX02 combination was superior to HLX02 and HLX11 combination. With these unique advantages over the HLX02 and HLX11 combination, the combination of HLX22 and HLX02 may prove to be of substantial benefit to gastric cancer patients who currently respond suboptimal to trastuzumab therapy. In clinical development, HLX22 was well tolerated at 3, 10, and 25 mg/kg once every 3 weeks in the phase I dose-escalation study aiming to evaluate the safety, pharmacokinetics, pharmacodynamics, and preliminary efficacy of HLX22 in patients with advanced solid tumors who had failed or were intolerant to standard therapies. No dose-limiting toxicity occurred during the study, and no serious adverse events occurred during the treatment period [14]. And further phase II trials to evaluate the clinical efficacy and safety of HLX22 in combination with HLX02 and chemotherapy in the HER2-positive locally advanced or metastatic gastric cancer as the first-line therapy are warranted.

### Supplementary Information

Additional File 1.

Additional File 2.

## Data Availability

The data needed to evaluate the conclusions in this study are available within the article and/or its supplementary data files. The RNA-sequencing data generated, and extended materials and methods in this study are available upon reasonable request from the corresponding author. Extended materials and methods are provided in the Additional file 2: Supplementary information.

## Abbreviations

ADCC: Antibody dependent cell-mediated cytotoxicity
AKT: Protein kinase B
BAD: BCL2 associated agonist of cell death
CDX: Cell line-derived xenograft
EGFR: Epidermal growth factor receptor
FGF: Fibroblast growth factors
FGFR: Fibroblast growth factor receptor
HER2: Human epidermal growth factor receptor 2
HIF1: Hypoxia-inducible factor 1
IgG1: Immunoglobulin G1
MTOR: Mammalian target of rapamycin
NCG: NOD/ShiLtJGpt-Prkdcem26Cd52Il2rgem26Cd22/Gpt
NJMU: Nanjing Medical University
PDX: Patient-derived xenograft
PI3K: Phosphoinositide 3-kinase
PLCG: Phospholipase C Gamma
RAF: Rapidly Accelerated Fibrosarcoma
RAS: Rat sarcoma
STAT3: Signal transducer and activator of transcription 3
STR: Short tandem repeats
TGF-β: Transforming growth factor-β
TPM: Transcript per million
