# Supplementary material for: Dual targeting non-overlapping epitopes in HER2 domain IV substantially enhanced HER2/HER2 homodimers and HER2/EGFR heterodimers internalization leading to potent antitumor activity in HER2-positive human gastric cancer
Source: J Transl Med. 2024 Jul 9;22:641. doi: 10.1186/s12967-024-05453-8 (PMC11232313; doi:10.1186/s12967-024-05453-8)

**Fig. S1 Variable region sequence of HLX22 light chain and heavy chain**

Variable region of Heavy Chain

1. EVQLVESGGG LVQPGGSLRL SCAASGFTFS SYTMSWVRQA PGKGLEWVAY 51 ISAGGGSTYY PDTVKGRFTI SRDNAKNSLY LQMNSLRAEDTAVYYCARHL

101 GGTASFDYWG QGTLVTVSS

Variable region of Light Chain

1. DIQMTQSPSS LSASVGDRVT ITCLASQTIG TWLAWYQQKP GKAPKLLIYV

51 ATSLADGVPS RFSGSGSGTD FTLTISSLQP EDFATYYCQQ NAYAPWTFGQ

101 GTKVEIK

**Fig.S2 HLX22 and HER2 ECD docking analysis by MOE.**

(**a**) HLX22 and trastuzumab have different binding epitope on HER2 ECD Domain IV. (**b**) binding sites between HLX22 and HER2 ECD.

a b


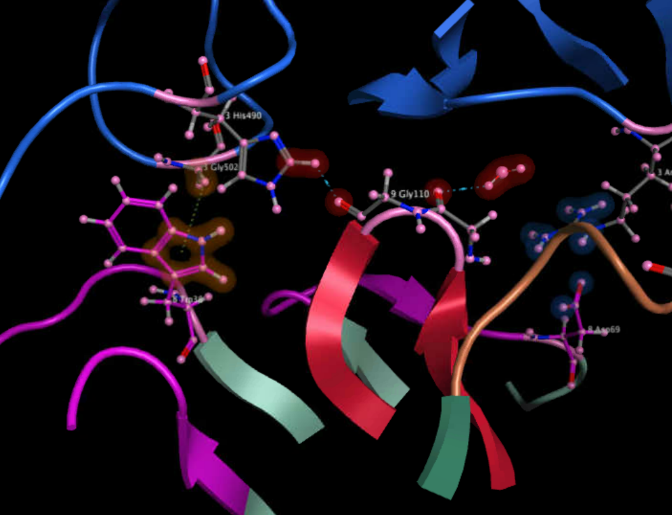

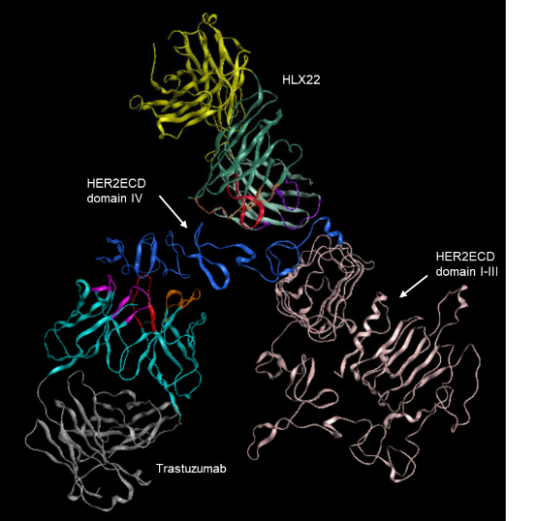


**Fig. S3 mRNA expression level of HER2, HER3 and EGFR in human stomach cancer or breast cancer.**

**a-c** mRNA expression level of HER2 (**a**), HER3 (**b**) and EGFR (**c**) in cancer category in TCGA database. TPM: Transcripts Per Million.


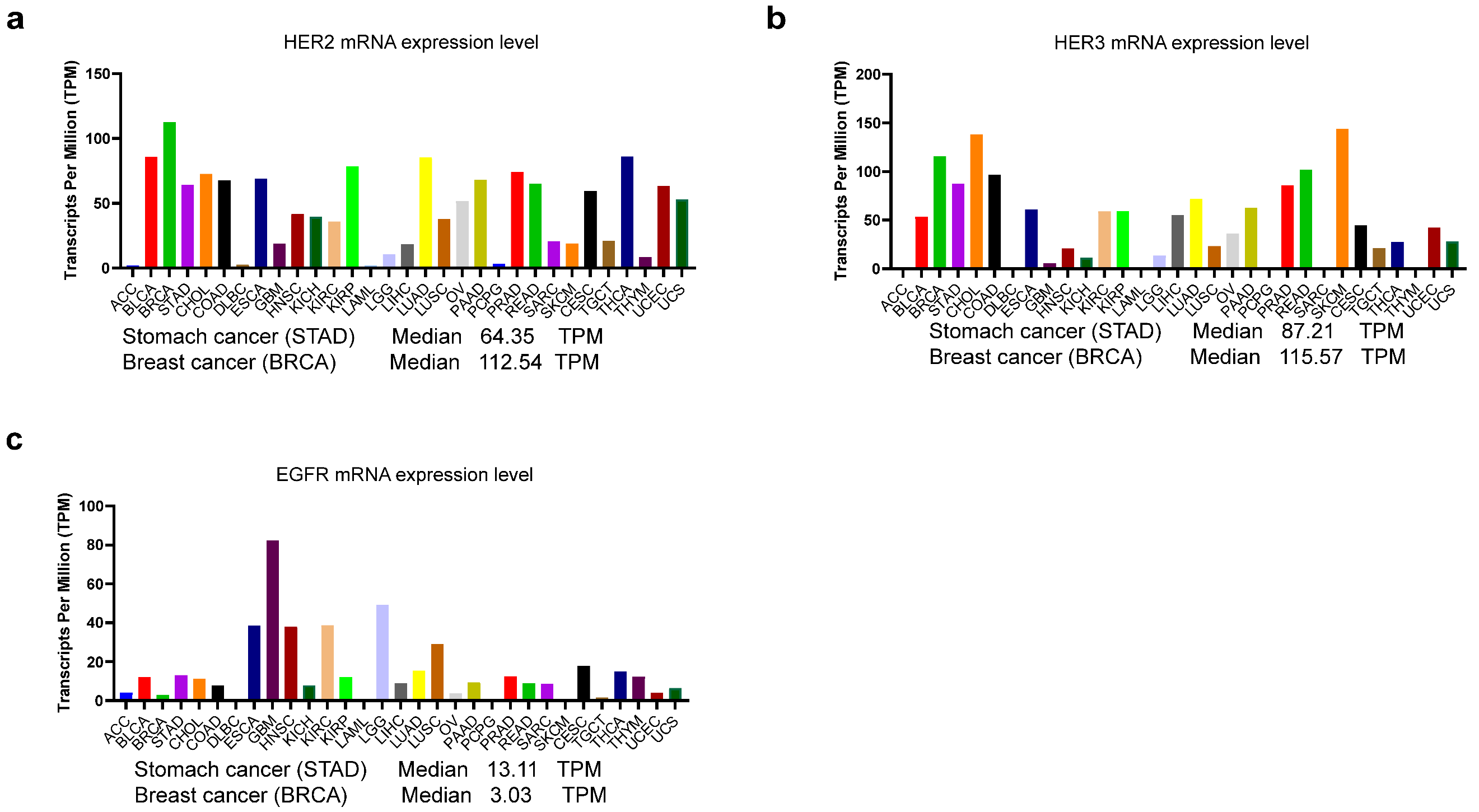


**Fig.S4 Impacts of EGFR expression on the efficacy of HLX22 and HLX02 combination.**

**(a)** The combination effects of HLX02 and HLX22 in EGFR knockdown N87 cells. (b) The combination effects of HLX02 and HLX22 in EGFR+++ and EGFR++ *in vitro* patient-derived tumor models


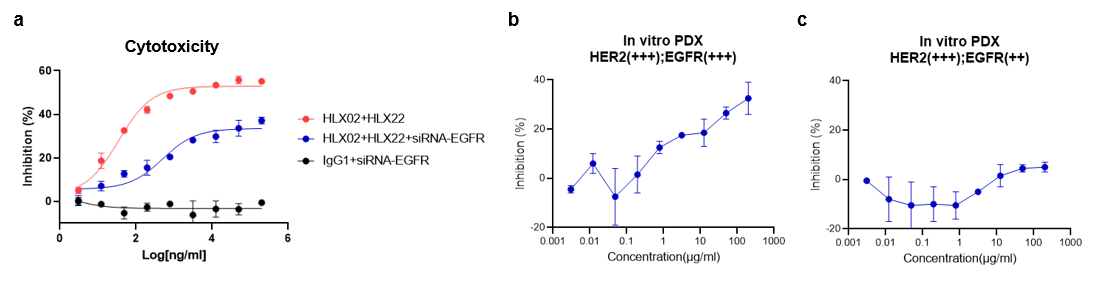

Supplement: Supplementary file 1 — Additional File 1. [file 12967_2024_5453_MOESM1_ESM.docx]
