# Supplementary material for: Dual targeting non-overlapping epitopes in HER2 domain IV substantially enhanced HER2/HER2 homodimers and HER2/EGFR heterodimers internalization leading to potent antitumor activity in HER2-positive human gastric cancer
Source: J Transl Med. 2024 Jul 9;22:641. doi: 10.1186/s12967-024-05453-8 (PMC11232313; doi:10.1186/s12967-024-05453-8)
Supplement: Supplementary file 2 — Additional File 2. [file 12967_2024_5453_MOESM2_ESM.pptx]

## Slide 1
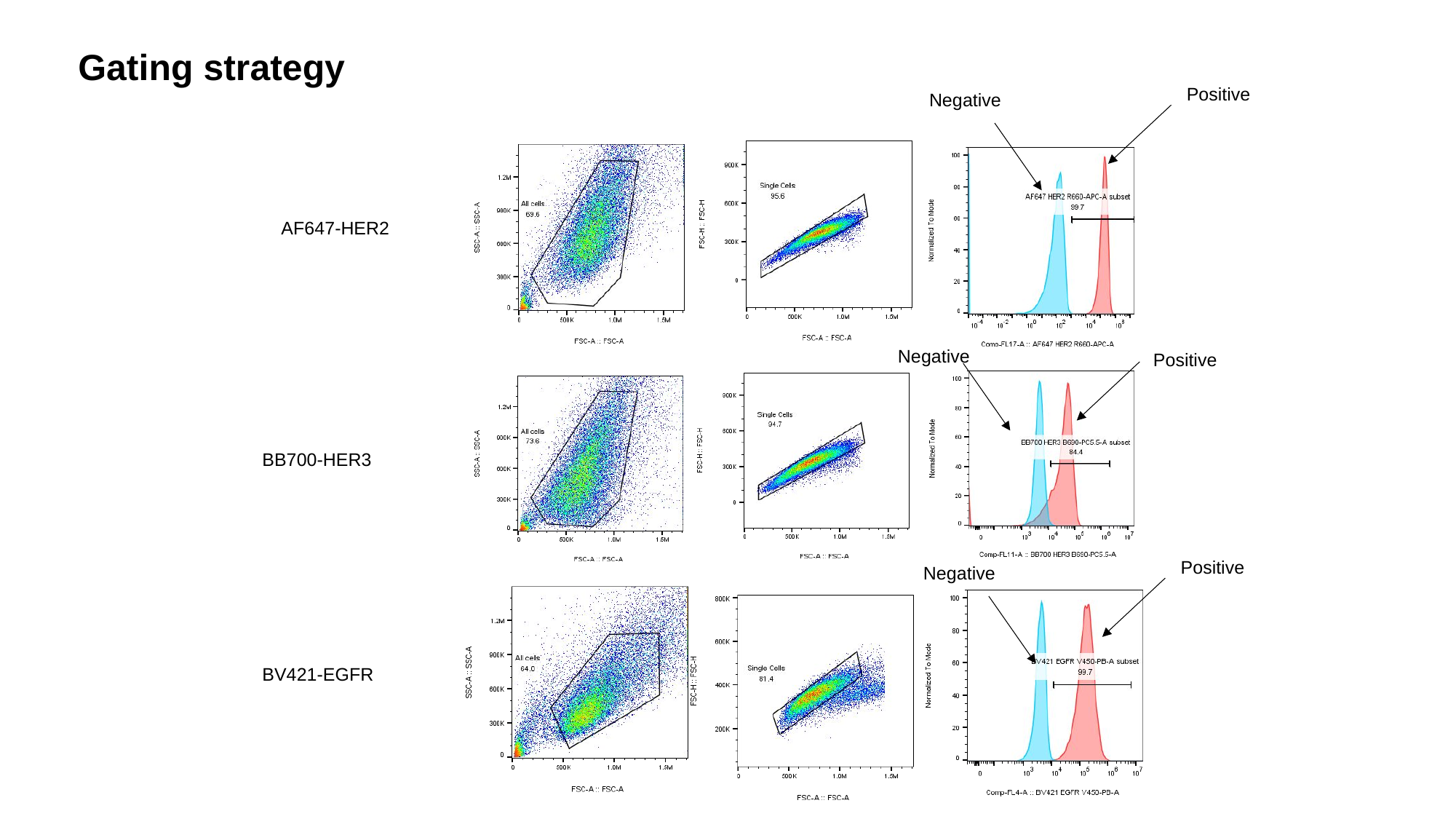

Gating strategy
Positive
Negative
AF647-HER2
Negative
Positive
BB700-HER3
Positive
Negative
BV421-EGFR
